# Electronic patient blood management monitoring using routine health record data: A proof‐of‐principle study monitoring perioperative tranexamic acid use

**DOI:** 10.1111/trf.70270

**Published:** 2026-05-19

**Authors:** Muhammad Naim Che Rahimi, Paolo Polzella, Mehir Amin, Gardash Bakhishli, Simon Godley, Michael F. Murphy, Simon J. Stanworth, Hayley G. Evans

**Affiliations:** ^1^ Department of Hematology Oxford University Hospitals NHS Foundation Trust Oxford UK; ^2^ ORBIT, Data & Analytics Oxford University Hospitals NHS Foundation Trust Oxford UK; ^3^ NIHR Blood & Transplant Research Unit in Data‐Driven Transfusion Practice Oxford UK; ^4^ Nuffield Division of Clinical Laboratory Sciences Radcliffe Department of Medicine, The University of Oxford Oxford UK

**Keywords:** audit, electronic health records, NICE quality standard, patient blood management, quality improvement, tranexamic acid

## Abstract

**Background:**

Monitoring Patient Blood Management (PBM) practices against evidence‐based standards is essential for quality improvement; however, current approaches are limited. In the UK, perioperative tranexamic acid (TXA) use is a national quality standard, yet monitoring relies on manual audit cycles that are resource‐intensive and limited in scope. We evaluated whether an audit could be automated using routinely collected electronic health record (EHR) data.

**Methods:**

We performed a retrospective study at a tertiary NHS center using linked perioperative and transfusion datasets. Automated compliance indicators were constructed using coded procedures (denominator) and digitally documented TXA administration from WHO Surgical Safety Checklists and electronic prescribing records (numerator). A structured validation framework assessed data extractability, completeness, denominator coverage, coding accuracy, and concordance between electronic sources. Outputs were assessed by specialty and procedure and compared with contemporaneous manual audit findings.

**Results:**

Between July–September 2025, 800 eligible procedures were identified. Comparison with an independent dataset demonstrated procedural coverage of 96.2% and miscoding rate of 3.9%. Overall automated TXA compliance was 86.3%. Concordance between WHO checklist and electronic prescription was 74.2%, with explainable discordance patterns. Substantial inter‐specialty variation was identified, ranging from 98.2% (trauma and orthopedics) to 0% (vascular surgery). Compared with October–December 2024, overall compliance increased by 7.6%.

**Discussion:**

Automated EHR‐based audit of perioperative TXA compliance is feasible and demonstrates good validity. Structured validation confirmed data reliability, and full‐population extraction revealed granular specialty‐ and procedure‐level variation, likely undetectable by manual audits, supporting its wider evaluation as a continuous PBM quality monitoring tool.

**Abbreviation**
AABB: Association for the Advancement of Blood & Biotherapies
BNF: British National Formulary
EPR: electronic patient record
NCABT: National Comparative Audit of Blood Transfusion
NHBST: NHS blood and transplant
NHS: National Health Service
NICE: National Institute for Health and Care Excellence
NIHR: National Institute for Health and Research
OPCS‐4: Office of Population Censuses and Surveys Classification of Surgical Operations and Procedures, 4th revision
OUH: Oxford University Hospitals
PBM: patient blood management
PBMPC HIC: patient blood management and perioperative care health informatics collaborative
QI: manual quality insights audit tool
QS138: NICE quality standard 138
SQL: structured query language
TXA: tranexamic acid
WHO: World Health Organization

## 1 INTRODUCTION

There is an expanding evidence base for many elements of patient blood management (PBM). Robust evidence for perioperative tranexamic acid (TXA) across orthopedics,^1^ cardiac,^2^ obstetric,^3^ and major abdominal^4^ surgery demonstrates that prophylactic perioperative TXA reduces bleeding^5^ and allogeneic transfusion exposure^6^ without increasing thromboembolic risk.^7^ The preliminary results from the TRACTION trial suggest these benefits extend to a hospital‐level policy of routine TXA use across major non‐cardiac surgery.^8^ TXA use in surgical procedures is recommended in multiple national as well as international guidelines.^9^, ^10^ In the UK, perioperative TXA administration is one of the four NICE transfusion quality standards, establishing it as a measurable indicator of institutional PBM performance.^11^

Monitoring of PBM practices is integral to quality improvement, yet in the context of TXA use, there is evidence that uptake of perioperative TXA use remains variable and sub‐optimal.^12^, ^13^ In the UK, current approaches to monitoring rely on manual data collection and retrospective case review, primarily undertaken through national annual audits of the NICE Quality Standard QS138^13^ and more frequent local and regional audit cycles using the QS138 Quality Insights (QI) tool.^14^ While valuable for benchmarking, these audits are resource‐intensive, limited in scale, and provide delayed feedback for quality improvement. A further constraint is sample size: sites typically contribute only 10 patients per audit cycle, introducing selection bias and limiting the ability to detect variation in practice across different specialties and procedures.

The digitization of perioperative and transfusion services creates the opportunity to use routinely collected electronic health record (EHR) data for large‐scale automated monitoring. In principle, such systems could enable comprehensive and timely assessment of perioperative TXA use. However, this potential remains underexplored in transfusion medicine, with perceived challenges including data governance, heterogeneous coding practices and variations in digital maturity across healthcare organizations.

The aim of this work was to design an automated TXA audit through integrated perioperative and transfusion data, and to provide an exemplar for other aspects of PBM. We developed a structured framework to evaluate its validity by comparison to the current standard of auditing, and to demonstrate how the routine collection of large amounts of data might reveal patterns of practice that are not readily observed through existing audit methodologies. This study demonstrates that a national PBM quality indicator can be automated using routinely collected electronic data and validated against linked routine data sources.

## 2 STUDY DESIGN AND METHODS

### 2.1 Study design and setting

We conducted a retrospective observational study undertaken at a single academic center, Oxford University Hospitals (OUH) NHS Foundation Trust, comprising four different hospital sites, using routinely collected electronic data. Two audit periods were examined: October–December 2024 and July–September 2025, coinciding with local participation in the national manual audit of the NICE QS138 standard for perioperative TXA. As this work formed part of service evaluation and quality improvement, formal research ethics approval was not required.

### 2.2 Data sources and electronic infrastructure

OUH uses an integrated EHR ecosystem incorporating Cerner Millennium, deployed across two separate instances spanning its hospital sites. Multiple perioperative, clinical, and transfusion‐related modules are relevant to this audit, alongside two transfusion systems – BloodTrack Tx for bedside transfusion records and Telepath, the transfusion laboratory information system. The SurgiNet module within Cerner Millennium provides structured operating theater documentation and includes the electronic World Health Organization (WHO) Surgical Safety Checklist with a mandatory field for prophylactic TXA administration. For analytical purposes, data from both Cerner instances are accessible through a unified Cerner Data Warehouse, a read‐only repository optimized for bulk querying that underpinned the SQL‐based data extraction for this study. These data flow into the Patient Blood Management and Perioperative Care (PBMPC) Health Informatics Collaborative (HIC) database.^15^ In brief, the PBMPC HIC database is a research database developed through the National Institute of Health and Research (NIHR) Health Informatics Collaborative, integrating routinely collected perioperative and transfusion data across participating NHS Trusts. At OUH, the PBMPC dataset includes structured procedural data extracted from the Data Warehouse and undergoes periodic validation against source systems. For this analysis, the PBMPC dataset was used as a reference standard for estimating denominator coverage and coding accuracy. As PBMPC data are subject to national data opt‐outs, true procedural volume may be marginally underestimated.

**FIGURE 1:**
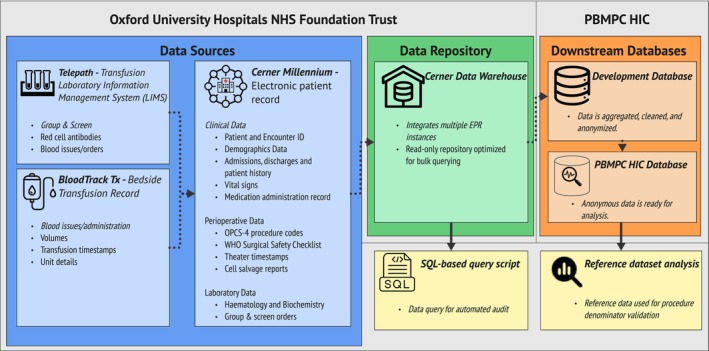
Overview of perioperative and transfusion data flows at Oxford University Hospitals (OUH) NHS Foundation Trust, including integration with the Patient Blood Management and Perioperative Care (PBMPC) Health Informatics Collaborative (HIC) database. OPCS‐4, Office of Population Censuses and Surveys Classification of Surgical Operations and Procedures, 4th revision; WHO, world health organization; PBMPC HIC, Patient Blood Management and Perioperative Care Health Informatics Collaborative.

A schematic overview of perioperative and transfusion data flows is shown in Figure 1.

### 2.3 Development of the automated QS138 audit and workflow

**FIGURE 2:**
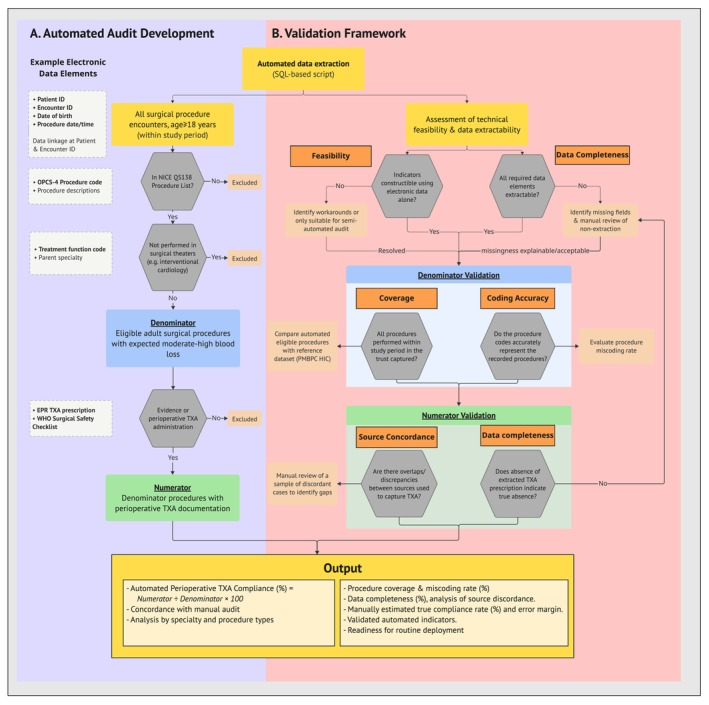
Automated perioperative tranexamic acid (TXA) audit development and validation. (A) Workflow to construct the NICE QS138‐based denominator (eligible adult surgical procedures) and numerator (procedures with perioperative TXA). (B) Validation framework assessing extractability and completeness of required data elements, denominator and numerator validation. EPR, electronic patient record; QS138, NICE Quality Standard 138; OPCS‐4, Office of Population Censuses and Surveys Classification of Surgical Operations and Procedures, 4th revision; TXA, tranexamic acid; WHO, world health organization.

The automated audit was designed to replicate the NICE QS138 audit using routinely collected digital data. The operational definitions for the denominator and numerator and adaptations made to align the automated audit with NICE QS138 are summarized in Table 1, alongside a comparison with the manual national audit. As this automated audit was developed prior to the recent update to the NICE guidance on perioperative TXA usage, the earlier NICE QS138 definition for the denominator was used, that is, surgical procedures with an expected moderate‐high risk blood loss (>500 mL). The automated logic used to construct the audit is illustrated in Figure 2A. Compliance was defined as the proportion of eligible procedures with documented perioperative TXA administration.

**TABLE 1:** Comparison of indicator definitions, data sources, and methodological characteristics across NICE QS138, the automated audit, and the manual Quality Insight audit for NICE Quality Standard QS138 Statement 2 (perioperative tranexamic use).

	NICE QS138 (pre‐2026 definition)	Manual quality insight audit	Automated audit
**Denominator**			
Definition	Adult (≥18 years) surgical procedures expected to have blood loss >500 mL	Adult (≥18 years) surgical procedures which are listed in the NICE QS138‐recommended OPCS‐4 procedure list; applied by local reviewer	Adult (≥18 years) surgical procedures which are listed in the NICE QS138‐recommended OPCS‐4 procedure list
Procedure identification	Clinical judgment at time of surgery; expected blood loss>500 mL documented on WHO Surgical Safety Checklist	Manual case note review; auditor selects cases from applicable procedure groups using NICE138 procedure list as guide	OPCS‐4 procedure code mapped to QS138 procedure list; non‐surgical and interventional cardiology encounters excluded via treatment function filter
Sample coverage	All eligible cases (national standard)	Minimum 10 cases per quarter; cases selected at random within the applicable patient group	All eligible cases per quarter (complete census)
Contraindication exclusion	No explicit exclusion stated	Cases where TXA is contraindicated per BNF guidance excluded as per auditor's review	Not applied; patient‐specific TXA contraindications cannot be reliably identified from structured electronic fields
**Numerator**			
Definition	Adults in the denominator who receive TXA	Adults in the denominator with evidence of TXA receipt identified on manual case review	Adults in the denominator with perioperative TXA documented digitally in either of two electronic sources
TXA identification	Any documented TXA administration perioperatively	Written or electronic prescription or administration record identified on manual review of case notes or EPR	WHO Surgical Safety Checklist field (“Tranexamic acid given = yes”) **or** EPR medication administration record on day of surgery
Handwritten records captured	Yes	Yes	No, TXA administration documented solely on handwritten anesthetic charts is not captured by electronic extraction.
Reactive intraoperative TXA included	Not explicitly addressed	Not explicitly addressed in audit guidance; dependent on individual auditor interpretation although Previous NCABT report provided breakdown of timing of TXA.	Provisionally included from EPR record; extent of misclassification examined in validation analysis
Data source	Not specified; determined locally – may include paper case notes, electronic notes, or both	Case notes, paper and/or electronic anesthetic and prescribing records	Cerner EPR, WHO Surgical Safety Checklist, electronic prescribing records

*Note*: NICE QS138 definitions reflect the pre‐February 2026 quality standard.

Abbreviations: BNF, British National Formulary; EPR, electronic patient record; NCABT, National Comparative Audit of Blood Transfusion; OPCS‐4, Office of Population Censuses and Surveys Classification of Surgical Operations and Procedures, 4th revision; QS138, NICE Quality Standard 138; TXA, tranexamic acid; WHO, World Health Organization.

Eligible surgical procedures (denominator) were identified by mapping Office of Population Censuses and Surveys Classification of Surgical Operations and Procedures 4th revision (OPCS‐4) procedure codes to the moderate blood loss surgery procedure list used in the QS138 Quality Insights audit guidance^14^—hereafter referred to as “QS138 procedure list” (Table S1). Procedures were identified at the level of individual theater encounters. Non‐surgical procedures, including interventional cardiology, were excluded by using the treatment function filters, which correspond to the parent specialty a procedure is performed under. Patients' date of birth was used to exclude individuals aged under 18 years.

Contraindications to TXA could not be reliably identified using structured electronic fields and were therefore not incorporated into exclusion criteria. National audit data suggest that thrombosis concerns and documented contraindications account for a small minority of cases where TXA is not administered (approximately 3%–5% of non‐compliant cases), though these figures are based on incomplete documentation and may underestimate true prevalence.^13^, ^16^

Perioperative TXA administration (numerator) was identified from two independent electronic sources: (1) the WHO Surgical Safety Checklist, and (2) electronic medication administration entries for TXA recorded on the day of surgery. Evidence from either source was counted as compliance to maximize capture of prophylactic TXA use. The degree of overlap and independence between these two sources was examined in the validation analysis. As the electronic prescribing records do not distinguish prophylactic at‐induction from reactive intraoperative TXA use, cases identified solely from EPR entries were provisionally classified as compliant. The extent of misclassification from this assumption was examined during the validation analysis.

### 2.4 Data elements and linkage

Data were extracted using SQL‐based scripts querying the Cerner Data Warehouse and linked at patient‐encounter level. Extracted elements included demographics, OPCS‐4 procedure codes and timestamps, specialty and treatment function, WHO checklist entries, electronic prescribing records, and transfusion data. Where multiple eligible OPCS‐4 codes were recorded within a single theater encounter, only the code at the primary procedure position was used to ensure each theater encounter was counted once in the denominator.

### 2.5 Validation framework

To assess the robustness and readiness of the automated audit workflow for routine use, we applied a structured validation framework adapted from Campbell's indicator testing protocol.^17^ The validation focused on two core questions:

First, whether all required data elements could be extracted reliably from routine electronic sources and whether the QS138 indicator of compliance could be generated without manual intervention; this included assessing extractability and completeness of key fields, mainly the WHO Checklist documentation and electronic prescribing records.

Second, whether the resulting indicators accurately represented the intended QS138 definitions. For the denominator, this involved evaluating coverage of eligible surgical procedures and the accuracy of OPCS‐4 coding. Coverage and coding accuracy were assessed by comparing automated OPCS‐4 coded procedure counts with the PBMPC HIC reference dataset, which contains free‐text procedure descriptions and therefore allowed identification of miscoded and under‐coded procedures.

For the numerator, we examined whether missing TXA records reflected true absence of administration or extraction failure, and assessed concordance between WHO Checklist documentation and electronic prescribing to determine how reliably each source captured prophylactic TXA use. Inter‐source concordance was assessed descriptively, and discordant cases were examined through structured manual review to characterize the clinical explanation for each discordant pattern.

These domains formed the basis of the structured field‐testing process summarized in Figure 2B.

### 2.6 Analysis plan

Automated outputs for each audit period were compared with contemporaneous manual QI audit findings, focusing on overall TXA compliance and magnitude of agreement. Given the descriptive, proof‐of‐principle design, analyses were primarily descriptive and no formal hypothesis testing was performed. Results were also examined by surgical specialty and procedure to identify variation in practice and potential targets for quality improvement.

## 3 RESULTS

### 3.1 Identification of automated denominators for procedures (coverage and coding accuracy)

In the July–September 2025 audit period, 965 OPCS‐4 coded procedures matching the QS138 procedure list were identified and extracted from the Cerner Data Warehouse across both OUH hospital instances. Following exclusion of 165 non‐surgical and interventional cardiology encounters, 800 eligible procedures were retained for the automated audit analysis.

Coverage and coding accuracy were assessed by comparison with the PBMPC HIC reference dataset, constructed by manual free‐text review of eligible surgical procedures performed in the same period. Eight hundred and thirty‐two eligible procedures were identified in the reference dataset, and when compared with 800 procedures captured by the automated audit, yielded an overall estimated denominator coverage of 96.2%.

Coding accuracy was assessed only among 798 procedures in the reference dataset that were assigned OPCS‐4 codes which are listed in the QS138 procedure list. This choice was made to reflect the operating conditions of the automated audit: these are the procedures the automated extraction captures when querying by QS138 OPCS code, and the miscoding rate therefore directly quantified the proportion of extracted procedures that may be misclassified at the point of capture. Of these 798 procedures, 31 (3.9%) were miscoded – assigned a QS138 OPCS‐4 code that did not correspond to the true procedure performed.

Collectively these findings indicate that automated extraction of procedures by OPCS‐4 codes provides high coverage with acceptable coding accuracy for the QS138 audit replication. Specialty‐level coverage and coding accuracy data are presented in Table 2.

**TABLE 2:** Specialty‐level denominator coverage and OPCS‐4 coding accuracy for NICE QS138 procedure list, comparing automated audit outputs with PBMPC HIC reference dataset (July–September 2025).

Specialty	Procedures in the automated audit (*n*)	Estimated Total procedures (*n*)^a^	Coverage (%)	Procedures coded with QS138 procedure OPCS codes (*n*)	Miscoded procedures (*n*)	Miscoding rate (%)^b^
Trauma & orthopedics	393	432	91.0	408	18	4.4
Cardiac Surgery	166	148	112.2^c^	153	8	5.2
Gynecology	107	97	110.3^c^	96	2	2.1
Colorectal	62	66	93.9	61	3	4.9
Urology	52	62	83.9	59	0	0.0
Vascular	20	27	74.1	21	0	0.0
Total	**800**	**832**	**96.2**	**798**	**31**	**3.9**

*Note*: The two sections are shaded differently to differentiate between the figures used for calculating procedural coverage and coding accuracy

Abbreviations: QS138, NICE Quality Standard 138; OPCS‐4, Office of Population Censuses and Surveys Classification of Surgical Operations and Procedures; PBMPC HIC, Patient Blood Management and Perioperative Care Health Informatics Collaborative.

^a^ Estimated total procedures include all eligible procedures identified by free‐text review of the PBMPC HIC reference dataset, encompassing those assigned QS138‐listed OPCS‐4 codes (correctly or incorrectly) and those assigned alternative OPCS‐4 codes outside the QS138 procedure list. True procedural volume may be marginally underestimated due to national data opt‐outs from the PBMPC HIC dataset.

^b^ Miscoding rate = miscoded procedures ÷ all procedures assigned a QS138‐listed OPCS‐4 code × 100. The denominator includes only OPCS‐4 codes listed in the QS138 procedure list, reflecting the pool of cases the automated audit captures when querying by OPCS codes, and therefore the rate at which the extracted procedures may be misclassified.

^c^ Coverage exceeds 100%, may be attributable to underestimation of true procedural volume in the PBMPC reference dataset due to national data opt‐outs.

### 3.2 Automated TXA compliance rate and comparison to manual audit

**FIGURE 3:**
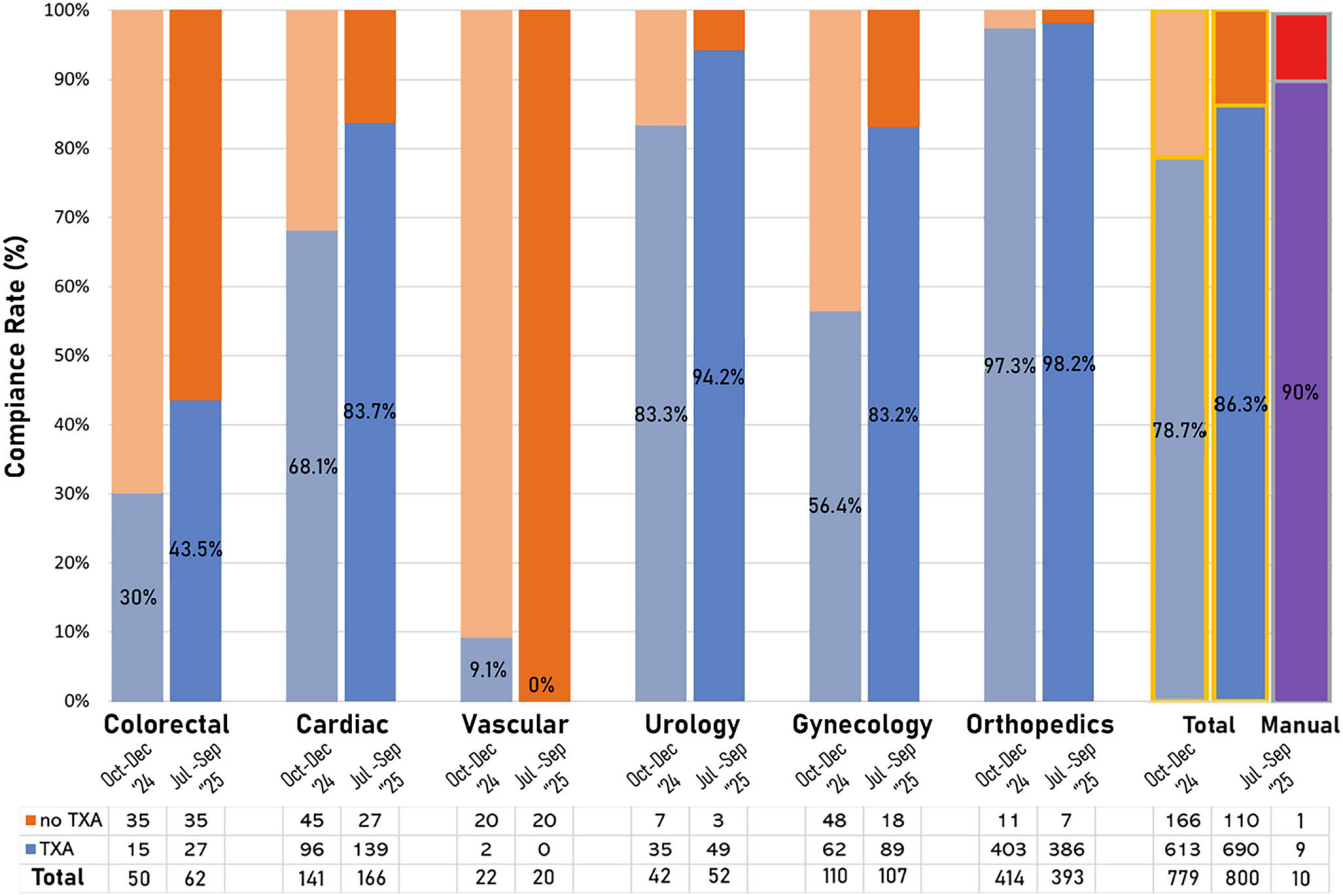
Perioperative tranexamic acid (TXA) compliance overall and by specialty using the automated audit workflow (NICE QS138 denominator list), with comparison between October–December 2024 and July–September 2025, and with manual audit undertaken in July–September 2025.

Between July and September 2025, the automated audit reported 86.3% overall perioperative TXA compliance comparable to the manual QI audit from the same period (90%, 10 cases) with significant inter‐specialty variation (Figure 3). Comparison with the October–December 2024 audit period revealed an overall compliance increase from 78.7% to 86.3%, with notable improvement in gynecology (+26.8%) and cardiac surgery (+15.6%).

### 3.3 Data extractability and completeness

Core numerator fields were reliably retrievable, and no manual review was required for the documentation of automated indicator of compliance. WHO Surgical Safety Checklist entries were extracted in 750 (93.8%) of eligible encounters; manual review of missing entries (*n* = 50) reflected mismatches in procedure and OPCS‐4 coding dates (*n* = 26, 52%), failure of linkage of extractable documents (*n* = 18, 12%) or, in maternity, the absence of mandatory TXA field on the WHO Checklist (*n* = 6, 2%). An electronic TXA administration record was present in 536 (67%) cases; manual review of a random sample of 50 non‐extracted cases confirmed true absence of electronic prescriptions rather than extraction failure. Thus, the observed “missingness” primarily reflects real‐world documentation practice with minimal technical loss.

### 3.4 Numerator definition fidelity and source concordance analysis

**FIGURE 4:**
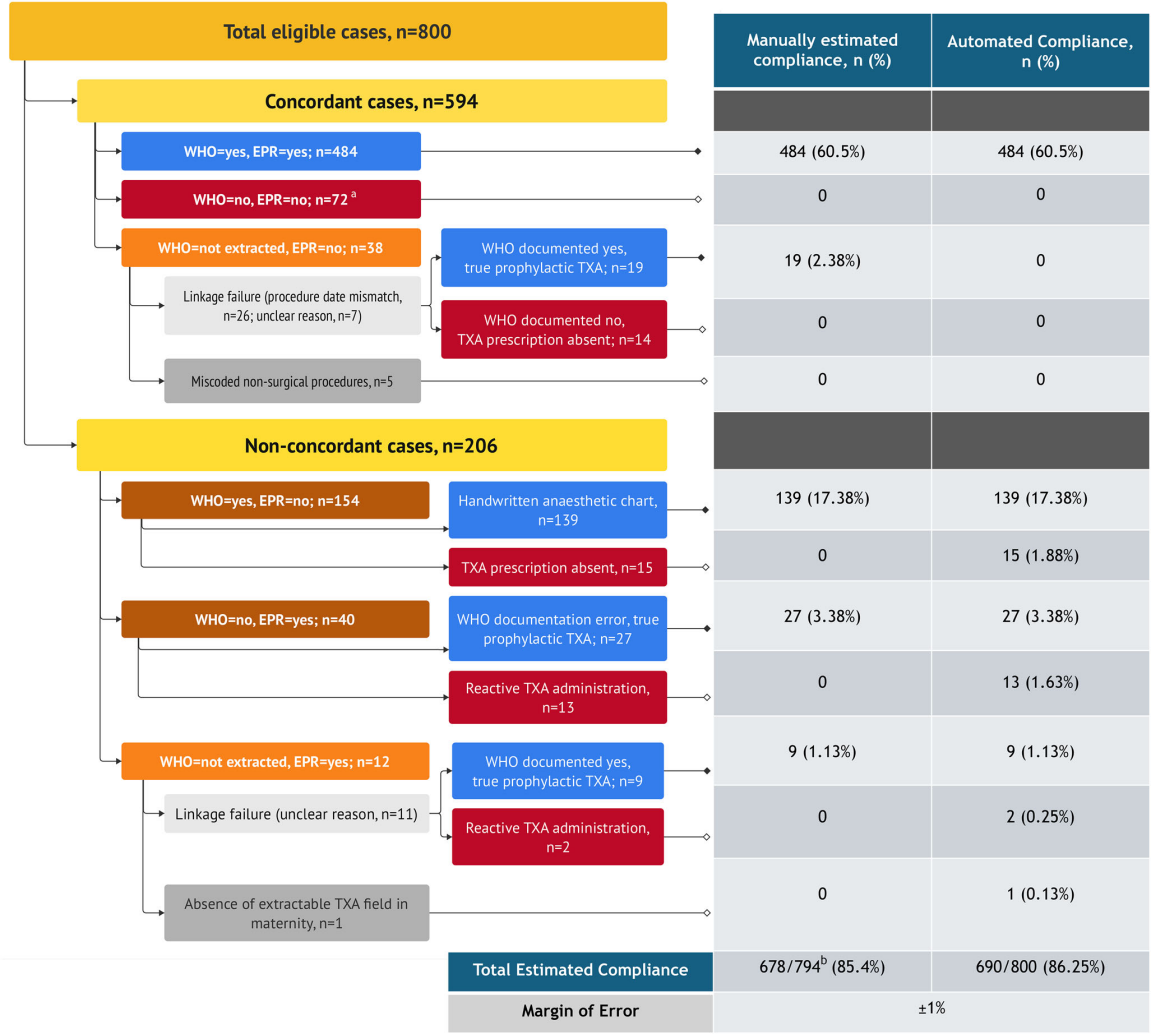
Case‐level concordance analysis of dual‐source perioperative TXA numerator construction (*n* = 800, July–September 2025). Flowchart illustrates the structured manual review of all 800 eligible procedures, comparing TXA identification between two electronic sources: The WHO Surgical Safety Checklist and the Electronic Patient Record (EPR). Cases are categorized as concordant (*n* = 594, 74.3%) or non‐concordant (*n* = 206, 25.8%), with full case‐level sub‐classification of each discordant group. The right panel presents a comparison of the automated dual‐source compliance estimate against the manually derived “true” compliance estimate following adjudication of discordant cases. (A) Manual review of 50 random WHO = no, EPR = no cases confirmed absence of EPR prescription rather than extraction failure. (B) Denominator for manual estimate is 794, excluding 5 miscoded non‐surgical procedures and 1 maternity case (absence of extractable TXA field). EPR, electronic patient record; TXA, tranexamic acid; WHO, World Health Organization Surgical Safety Checklist.

We evaluated the concordance between the two electronic sources used to identify perioperative TXA administration (numerator). Observed agreement between the WHO Surgical Safety Checklist and EPR was 74.2%, indicating moderate concordance. Disagreement between sources was asymmetric, with the WHO Checklist more frequently recording TXA than EPR entries (154 vs. 52 discordant cases). Structured manual review demonstrated this asymmetry was systematic and explicable: WHO = yes/EPR = no cases (*n* = 154) predominantly reflected continued use of handwritten anesthetic charts in cardiac surgery (*n* = 139); the remainder reflected absent EPR prescriptions. Among WHO = no/EPR = no cases (*n* = 52), three explanatory categories were identified: reactive intraoperative TXA administration, WHO documentation error in confirmed prophylactic cases, and extraction failure; the case‐level breakdown is illustrated in Figure 4.

Taken together, these findings confirmed acceptable data completeness, clarified the strengths and limitations of each source, and supported the validity of the dual‐source approach for automated perioperative TXA numerator documentation. The review process also allowed estimation of a “true” compliance rate of 85.3%, within 1% of the automated estimate.

### 3.5 High‐resolution variation in perioperative TXA use

**FIGURE 5:**
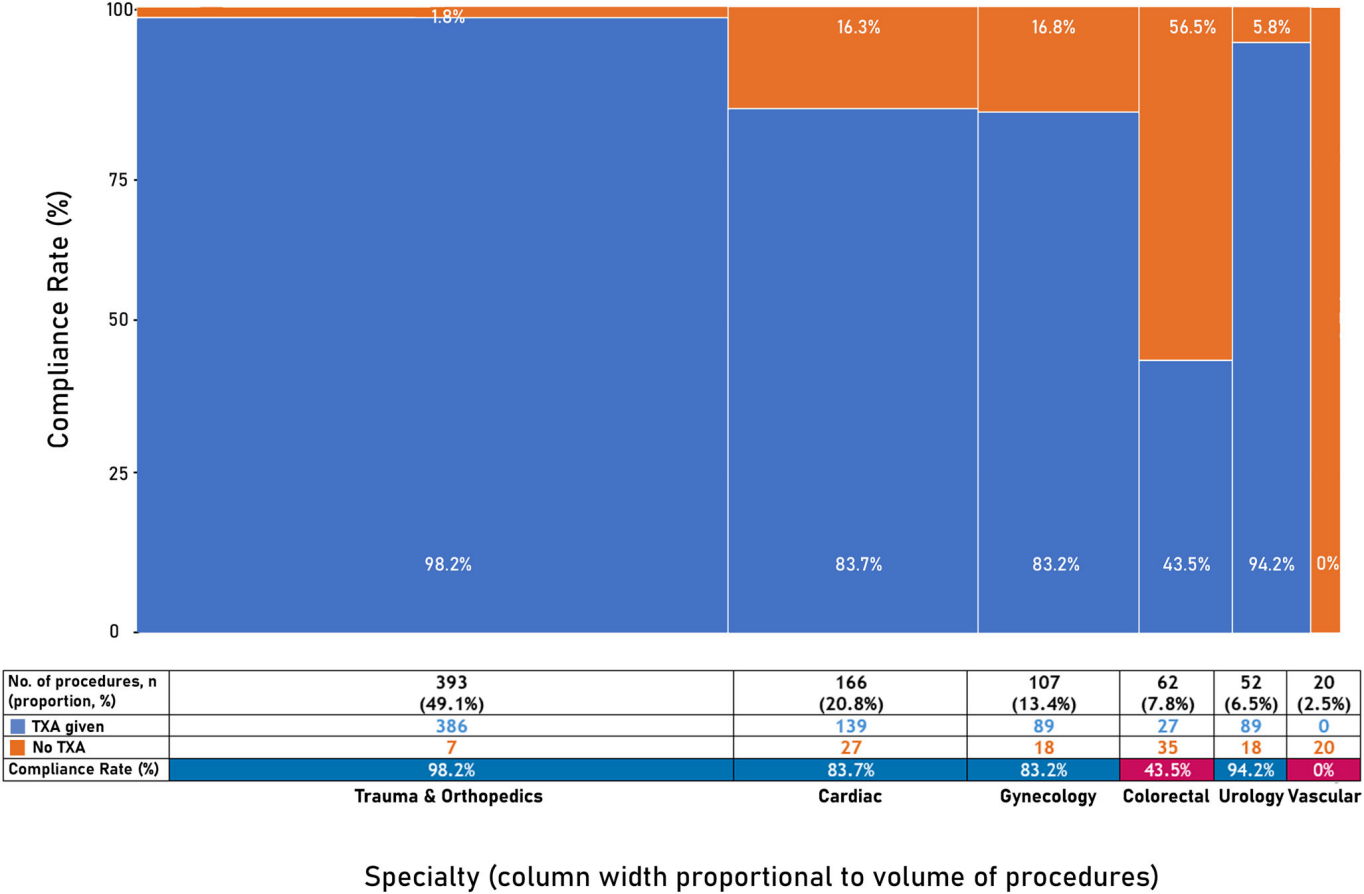
Mosaic chart showing perioperative TXA compliance by specialty (*n* = 800, July–September 2025). Column width is proportional to procedure volume. Specialties are ordered by descending procedure volume. TXA, tranexamic acid.

**FIGURE 6:**
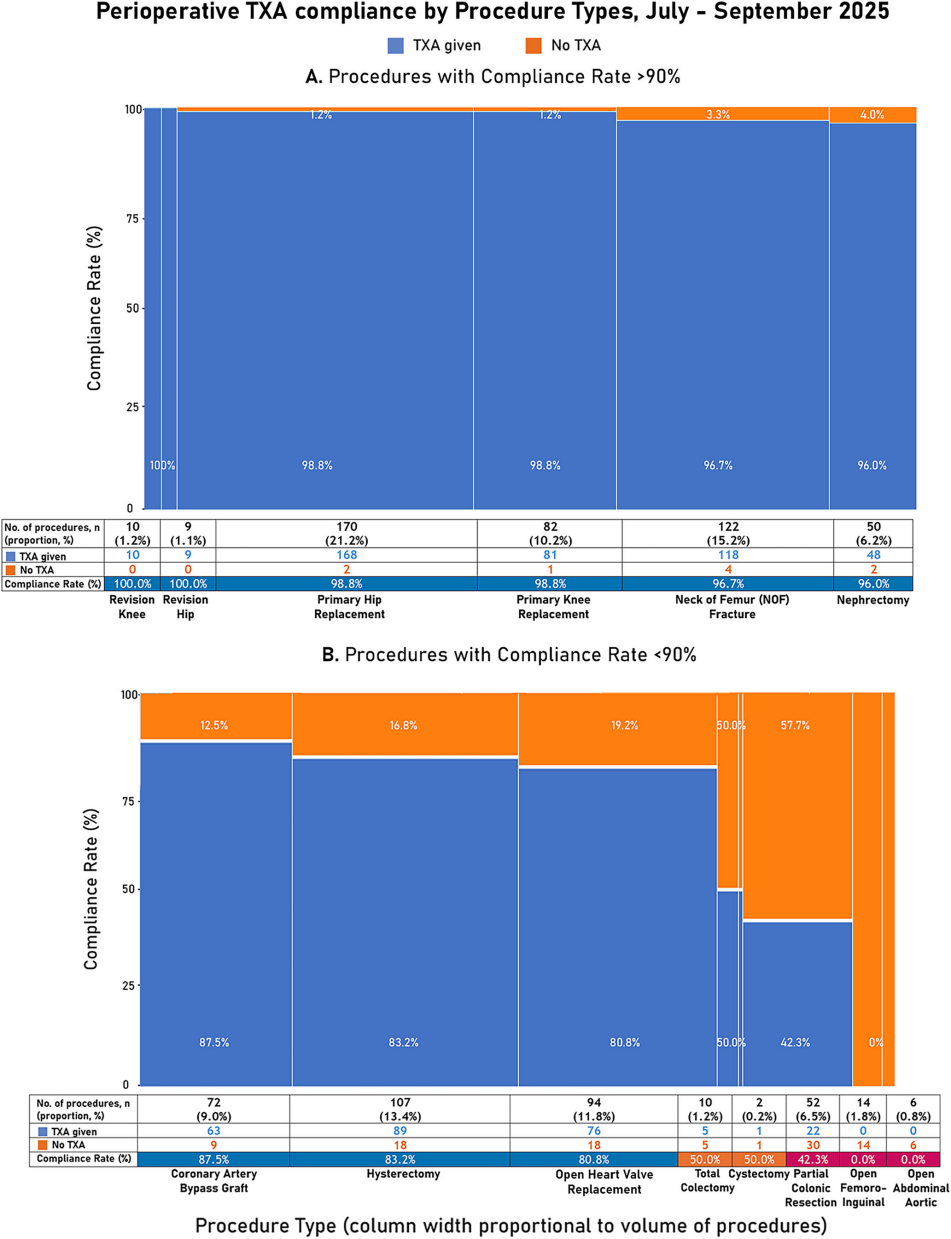
Mosaic charts showing perioperative TXA compliance rate by procedure type (*n* = 800, July–September 2025). Column width is proportional to procedure volume. Procedures are ordered by descending compliance rate and divided into two panels for visual clarity (A) Procedures with compliance >90%. (B) Procedures with compliance <90%. TXA, tranexamic acid.

The scale of the automated audit enabled specialty‐ and procedure‐level compliance analysis that would not have been feasible through conventional manual methods. To contextualize this variation by the relative procedural burden of each specialty and procedure type, compliance rates were assessed and visualized alongside volume using variable‐width column (Mosaic) charts, in which column width is proportional to the number of eligible procedures performed in the audit period (Figures 5 and 6A,B).

At the specialty level (Figure 5), trauma and orthopedics accounted for the largest procedural volume (*n* = 393; 49.1% of the total cohort) whilst maintaining the highest compliance rate (98.2%). Cardiac surgery represented the second largest volume (*n* = 166; 20.8%) with a compliance rate of 83.7%. Gynecology achieved 83.2% compliance (*n* = 107, 13.4%) and urology 94.2% (*n* = 52; 6.5%). Lower compliance rates were seen in colorectal at 43.5% (*n* = 62; 7.8%), and vascular surgery at 0% (*n* = 20, 2.5%).

At the procedure level (Figure 6A,B), higher compliance was seen in revision hip and knee replacements (100%), primary hip and knee replacements (98.8%), neck of femur fracture fixation (96.7%), nephrectomy (96.0%), coronary artery bypass grafting (87.5%), hysterectomy (83.2%), and open valve replacements (80.8%). Lower compliance was seen in the remaining procedures. Colorectal resections demonstrated the largest absolute shortfall, with 40 missed TXA administrations from 62 eligible procedures, reflecting its combination of low compliance and relatively high procedural volume. Open iliofemoral (*n* = 14) and abdominal aortic operations (*n* = 6) demonstrated 0% TXA compliance, and total colectomy (*n* = 10) and cystectomy (*n* = 2) both showed intermediate compliance at 50%. Procedural volumes across these groups were small, limiting reliability of these estimates and warranting further specialty‐level review. Taken together, these data reveal real‐world inter‐procedural variation in the use of perioperative TXA, and the volume‐weighted visualization allows identification of procedures where improvement in compliance may have the greatest absolute impact on perioperative TXA delivery.

## 4 DISCUSSION

As PBM moves from evidence generation to implementation, robust monitoring mechanisms are required, but the current audit approaches are manual and resource‐intensive. This proof‐of‐principle study demonstrates that assessment of compliance with a national quality standard for the use of perioperative TXA can be delivered using routinely collected EHR data. Automated identification of eligible procedures and extraction of TXA documentation were feasible without manual case review, and compliance estimates were comparable to contemporaneous manual‐based national audit sampling.

A key strength of this study is the structured validation of data elements underpinning the indicator of compliance. We examined denominator coverage, OPCS‐4 coding accuracy, completeness of numerator fields, and concordance between independent electronic sources of TXA documentation. High coverage relative to reference data suggests that procedure coding within our institution is sufficiently robust to support automated audit. Discordance between WHO checklist documentation and electronic prescribing was largely explicable by established workflow variation, including continued use of handwritten anesthetic charts in some specialties, rather than extraction failure. Manual adjudication further demonstrated that this source of potential numerator misclassification had minimal impact on the overall compliance estimate (<1%). Without such scrutiny, automated outputs risk being technically efficient but conceptually fragile.

Several limitations apply. The validation process exposes the documentation gaps on which the automated monitoring depends. Incomplete WHO TXA fields, inconsistent electronic prescribing, and specialty‐level reliance on paper records are revealed alongside compliance rates themselves. In this sense, automated audit functions not only as a performance measure but as a diagnostic tool for digital workflow optimization. National policy initiatives, including recommendations arising from the Infected Blood Inquiry, have emphasized the need for coherent and integrated transfusion data systems.^18^ The present findings illustrate both the feasibility of such integration and the necessity of systematic validation before outputs are used for benchmarking, particularly when cross‐site comparison is intended, where differences in automated audit outputs may reflect variation in documentation practices or digital maturity as much as true differences in PBM performance. Our evaluation study was conducted in a single tertiary healthcare organization with relatively mature digital infrastructure; performance may differ substantially in institutions with alternative EHR configurations, less comprehensive linkage, or variable coding practices. The validation framework described here provides a template for how such assessment could be undertaken at other sites.

Further limitations relate to the accuracy of the denominator and numerator. Contraindications to TXA could not be reliably identified from structured fields and were not incorporated into exclusion criteria. While national audit data suggest these account for only approximately 3–5% non‐compliant cases,^13^, ^16^ which may be an underestimate as they rely on incomplete data, their omission may result in systematic overestimation of non‐compliance in a minority of patients, which is an important caveat for benchmarking. Electronic prescribing records did not consistently distinguish prophylactic from reactive TXA administration, introducing the possibility of limited numerator misclassification. However, our structured manual review estimated a true compliance rate of 85.3%, within 1% of the automated figure, suggesting the practical impact of this misclassification was modest in our setting. Nonetheless, this remains an inherent limitation, and future iterations of the audit could seek to refine numerator classification by incorporating timestamp data to better distinguish prophylactic from reactive TXA administration. Additionally, due to the small sample size in the manual audit, comparison with the automated audit was descriptive only. This study is therefore best interpreted as demonstrating technical feasibility rather than establishing accuracy or superiority over existing methods. Future work should prioritize multicenter validation with larger comparative datasets to establish the accuracy and generalizability of the approach, and ideally linkage to clinical outcomes to determine whether improved monitoring translates into improved patient care.

Beyond measurement accuracy, this audit workflow is intentionally scoped to replicate the binary outcome specified by NICE QS138 (i.e., whether TXA was administered or not), and does not assess whether administration was consistent with evidence‐based protocols with respect to dose, timing relative to incision, or infusion regimens for longer procedures. Extended automated monitoring to capture these dimensions represents an important next step toward a more clinically complete assessment of PBM performance. Furthermore, the current workflow was designed to replicate the NICE QS138 OPCS‐4 based denominator, which does not currently encompass all surgical contexts in which TXA may be of benefit. Procedures associated with significant perioperative blood loss but not captured within the current list, such as spinal and obstetric procedures, represent an important gap in automated PBM surveillance coverage. Since this audit was developed, NICE has published updated guidance on perioperative TXA use,^10^ which substantially expands the recommended denominator; rather than restricting TXA to procedures with expected blood loss >500 mL, the updated guidance recommends offering TXA to all adults undergoing surgery in an operating theater where there is a risk of bleeding and the procedure breaches the mucous membrane. This represents a fundamental change in the scope of TXA compliance monitoring. Adapting the automated audit workflow to reflect this new denominator definition and evaluating the technical feasibility of doing so at scale represents both an immediate priority and an important area of future work. Future work should focus on multi center validation, integration with real‐time dashboards, and extension to other PBM indicators.

In summary, a procedure‐based audit of a PBM quality standard, developed in the context of manual audit, can be adapted and implemented reliably within routine digital systems. Automated audit addresses recognized limitations of manual sampling in most current audits. In the UK, typically small samples are collected quarterly across multiple hospital sites for auditing, but these are inherently imprecise and may obscure important variation within specialties or procedures. Full‐population extraction that is only possible with more electronic capabilities provides more stable estimates and enables granular analysis at specialty and procedure level. An electronic approach can also better facilitate change in practice. The effectiveness of audit and feedback is influenced by clinicians' perception of data credibility and relevance.^19^, ^20^, ^21^ Feedback that reflects local case mix and permits like‐for‐like comparison is more likely to drive engagement than aggregated benchmarks. Automated extraction provides this specificity without proportionate increase in administrative burden. However, establishing minimum standards of digital readiness and transparent reporting of data quality assumptions will be essential if automated PBM surveillance is to be expanded and implemented to other sites and used for credible benchmarking. With appropriate validation, such infrastructure could move PBM monitoring from intermittent retrospective audit toward continuous, data‐driven quality improvement at scale.

## AUTHOR CONTRIBUTIONS

M.CR. and P.P. contributed equally to this work and share first authorship; M.M, S.S, and H.E. contributed equally as senior authors. P.P and M.A. designed the automated audit workflow; M.A. developed the code for the data extraction script; G.B. processed the reference data from PBMPC HIC database; M.CR. collated, reviewed, and analyzed the data; S.G and H.E. supported the audit project; M.CR. and H.E. drafted of the manuscript. All authors were involved in the design and conduct of the study and reviewed and updated the manuscript.

## FUNDING INFORMATION

This project is supported (in part) by the UK NIHR Blood and Transplant Research Unit in Data Driven Transfusion Practice (NIHR203334).

## CONFLICT OF INTEREST STATEMENT

The authors have disclosed no conflicts of interest.

## Supporting information


**Table S1.** Surgical procedure list and corresponding OPCS‐4 codes used as the denominator for the automated audit. Adapted from QS138 Quality Insights Audit Tool User Guide.

## Data Availability

The data that support the findings of this study are available from the corresponding author upon reasonable request.
