# Supplementary material for: Electronic patient blood management monitoring using routine health record data: A proof‐of‐principle study monitoring perioperative tranexamic acid use
Source: Transfusion. 2026 May 19;66(8):1506–19. doi: 10.1111/trf.70270 (PMC13452614; doi:10.1111/trf.70270)
Supplement: Supplementary file 1 [file TRF-66-1506-s001.docx]

**SUPPLEMENTARY TABLES**

**Supplementary Table S1**. Surgical procedure list and corresponding OPCS-4 codes used as the denominator for the automated audit. Adapted from QS138 Quality Insights Audit Tool User Guide.

| **Surgical Specialty** | **Procedure Type** | **OPCS-4 Codes** |
| --- | --- | --- |
| Trauma & Orthopedics | Hip replacement (primary & revision) | W371/3/4, W381-W384, W393-W395, W93, W941-W944 |
|  | Knee replacement (primary & revision) | W401-W403, W423/4 |
|  | Neck of femur fracture arthroplasty | W191, W241/6, W396, W461/3 |
| Cardiac Surgery | Open valvular procedures | K25, K26, K27, K28 |
|  | Coronary arterial bypass grafts | K40 – K46 |
| Gynecology | Hysterectomy | Q07, Q08 |
| Colorectal Surgery | Total & subtotal colectomy | H05, H29 |
|  | Partial colorectal resections & proctectomy | H06, H07, H33, H41 |
| Vascular Surgery | Open abdominal aortic procedures | L18, L19, L20, L21 |
|  | Open ilio-femoral artery procedures | L51, L59, L62 |
| Urology | Nephrectomy | M02, M03 |
|  | Cystectomy | M34, M35 |
